# Supplementary material for: Preliminary Psychometric Evaluation of Novel Measures of Therapist Practice Related to LGBTQ+ Clients
Source: Healthcare (Basel). 2024 Jan 3;12(1):110. doi: 10.3390/healthcare12010110 (PMC10779343; doi:10.3390/healthcare12010110)
Supplement: Supplementary file 1 [file healthcare-12-00110-s001.zip › healthcare-2741646-supplementary.pdf]

SCHOOL OF PUBLIC HEALTH
PREVENTION RESEARCH CENTER

Mental Health Therapist LGBTQ+ Competency Self-Assessment

The Mental Health Therapist LGBTQ+ Competency Self-Assessment was created by the University of Maryland Prevention Research Center for mental and behavioral health professionals to self-reflect and assess their own competencies related to providing LGBTQ+ affirmative care in the following areas: knowledge, attitudes, self-efficacy, and practice.

This self-assessment takes 15-20 minutes to complete. Each section includes scoring information and interpretations to assist professionals in understanding their current levels of LGBTQ+ competencies.

For additional training resources and information, please visit the UMD-PRC Resources Page and consider participating in the UMD-PRC Sexual and Gender Diversity Learning Community Certificate Program.

KNOWLEDGE

Please indicate where on the scale you would rate the following items:

0 = Completely false
1 = Probably false
2 = Probably true
3 = Completely true

_____ 1. Sexual orientation may evolve and change over the life course.
_____ 2. Physical sexual urges, romantic affections, and actual sexual behaviors must all be consistently directed toward people of the same sex for someone to be considered gay.
_____ 3. Gender identity is defined by the client, not based on expression or roles.
_____ 4. Gender identity may extend beyond man and woman.
_____ 5. *Having a gender identity that differs from sex assigned at birth constitutes a mental illness diagnosis.
_____ 6. Youth with gender dysphoria may either adopt a transgender or cisgender identity as adults.
_____ 7. Gender dysphoria is different than gender non-conformity.
_____ 8. Attempts to change a client's sexual orientation have negative effects on their mental health.
_____ 9. Attempts to change a client's gender identity have negative effects on their mental health.
_____ 10. Transgender persons may consider themselves straight, gay or some other sexual orientation.
_____ 11. Therapist use of client pronouns is directly related to client trust and comfort.
_____ 12. LGBTQ+ individuals' mental health problems are often due to lack of social acceptance.
_____ 13. The LGBTQ+ population experiences higher rates of substance use, sexually transmitted infections including HIV, and mental health challenges than the straight cisgender population.
_____ 14. The mental health concerns of LGBTQ+ persons may be associated with other marginalized identities (i.e. race, ethnicity, nativity) and not necessarily their LGBTQ+ status.

Knowledge Scoring Information

*Item 5 is reverse coded (where completely false = 3 and completely true = 0)

Add all 14 items above (ranges from 0 – 42) = ________

Interpretation: The higher your score, the higher your knowledge around LGBTQ+ issues.

Copyright © 2023 Bradley O. Boekeloo, Jessica N. Fish, Elizabeth M. Aparicio, Richard Shin, Mia Smith-Bynum, Michael Vigorito, & Sean Lare. University of Maryland Prevention Research Center.

ATTITUDES

Please indicate your level of agreement with the following items on a scale from:

0 = Strongly Disagree
1 = Somewhat Disagree
2 = Somewhat Agree
3 = Strongly Agree

_____ 1. *Obtaining more training regarding competence with LGBTQ+ clients isn't a good use of my time as a clinician.
_____ 2. I have received adequate clinical training and supervision to counsel lesbian, gay, and bisexual clients.
_____ 3. I have received adequate clinical training and supervision to counsel transgender and gender diverse clients.
_____ 4. *Same sex/gender attraction, affection, and behaviors are unnatural.
_____ 5. I know the definitions and common experiences of diverse sexual orientation identities including pansexual, asexual, and queer.
_____ 6. I understand the differences between a client's physical sexual attraction, romantic affection, actual sexual behavior, and identity related to sexual orientation.
_____ 7. I know what the terms transgender man, transgender woman, non-binary, and cisgender all mean.
_____ 8. *I believe that if transgender people would just accept their sex assigned at birth as their gender identity, they would be a lot happier.
_____ 9. Attempts to change an LGBTQ+ client's sexual orientation are unethical.
_____ 10. *I believe that clients who do not identify with their sex assigned at birth are a threat to natural order and that therapy should not reinforce this.
_____ 11. *I believe that clients who are not heterosexual are a threat to natural order and that therapy should not reinforce this.
_____ 12. *It is unrealistic for clients to expect their provider to always use names and pronouns that differ from their legal names and pronouns.
_____ 13. *It is OK for healthcare forms to be missing options for clients to identify as LGBTQ.
_____ 14. Society benefits from LGBTQ+ persons' perspectives on gender and sexuality.
_____ 15. It is important to speak up when LGBTQ+ persons are demeaned.
_____ 16. *LGBTQ+ people are inherently self-destructive.
_____ 17. *LGBTQ+ people make up a small percentage of the population so healthcare services cannot be expected to alter their services for this population.
_____ 18. I want my own practice to be known as LGBTQ+ affirming.
_____ 19. To me, referral involves not just providing referral contact information but also active client support in obtaining the services.
_____ 20. *Bias toward minority groups like Black LGBTQ+ persons is minimal in this day and age.
_____ 21. *Monogamy should be the ideal in all LGBTQ+ romantic relationships.
_____ 22. *It is never acceptable in mental health counseling to try and persuade parents with anti-LGBT religious beliefs to accept their children.
_____ 23. *In this day and age, LGBTQ+ people make too big of a deal of the coming out process.
_____ 24. *I believe mental health care providers should be able to withhold care for LGBTQ+ clients due to religious beliefs.
_____ 25. *I believe that children are best raised in households with a mother and father.
_____ 26. *I do not believe that transgender persons' unique healthcare expenses should be paid for with tax-payers money.

Attitude Scoring Information

*Reverse code items 1, 4, 8, 10-13, 16-17, 20-26 (where strongly disagree = 3 and strongly agree = 0)

Add all 26 items above (ranges from 0 – 78) = ________

Interpretation: The higher your score, the more positive are your attitudes towards LGBTQ+ persons.

Copyright © 2023 Bradley O. Boekeloo, Jessica N. Fish, Elizabeth M. Aparicio, Richard Shin, Mia Smith-Bynum, Michael Vigorito, & Sean Lare. University of Maryland Prevention Research Center.

SELF-EFFICACY

Please indicate your level of agreement with the following items on a scale from:

0 = Strongly Disagree
1 = Somewhat Disagree
2 = Somewhat Agree
3 = Strongly Agree

_____ 1. I am confident that I can obtain the training I need to improve my competence with LGBTQ+ clients.
_____ 2. I am confident that I know which skills that I need to focus on with more training to provide competent LGBTQ+ mental healthcare.
_____ 3. I am comfortable avoiding assumptions about clients' physical sexual drives, romantic affections, or actual sexual behaviors based on their self-identified sexual orientation.
_____ 4. I am confident that I can help clients navigate lack of alignment in their physical sexual urges, romantic affections, and sexual behaviors.
_____ 5. I am confident that I can provide appropriate counseling to pansexual persons.
_____ 6. I am confident that I can provide appropriate counseling to LGBTQ+ persons who are not monogamous.
_____ 7. I am confident that I can avoid making assumptions about clients' gender identity based on their outward gender expression.
_____ 8. I am confident that I can avoid making assumptions about clients' sex assigned at birth based on their gender identity.
_____ 9. I am confident that I can provide appropriate counseling to LGBTQ+ persons who identify their gender as non-binary.
_____ 10. I am comfortable not assuming clients' sexual orientation based on their gender identity.
_____ 11. I am comfortable not assuming clients' gender identity based on their sexual orientation.
_____ 12. I am confident that I can approach all LGBTQ+ clients in a way that will not exacerbate any past oppression.
_____ 13. I know what to do when I misgender a client.
_____ 14. I am comfortable using pronouns outside of the he/she binary such as they/them, zi/zir, and hir/hirs.
_____ 15. I am comfortable giving my pronouns when I introduce myself to clients.
_____ 16. Identifying my client's preferred name and pronouns is easy for me.
_____ 17. I am confident that I am able to help LGBTQ+ clients identify and build on their strengths and resources.
_____ 18. I am confident that I can help LGBTQ+ clients identify their own internalized homophobia, biphobia, and/or transphobia.
_____ 19. I am confident that I can help clients identify external sources of their internalized homophobia, biphobia, and/or transphobia.
_____ 20. I am confident that I can recognize ways in which my own practice might not be helpful to LGBTQ+ clients.
_____ 21. I am confident that I will speak up in my organization when I see things that may demean LGBTQ+ clients.
_____ 22. I am comfortable discussing sexual behaviors with LGBTQ+ clients.
_____ 23. I am comfortable discussing ways to prevent sexually transmitted infection like HIV with same gender couples.
_____ 24. I am confident that I can examine potential behavioral, social and medical referrals to make sure they are supportive of LGBTQ+ persons.
_____ 25. I am comfortable actively assisting LGBTQ+ clients in obtaining needed referral services.
_____ 26. I am comfortable talking about racial discrimination in the LGBTQ+ community.
_____ 27. When desired by LGBTQ+ clients, I am confident that I can explore with them the impact of racism on their mental health.
_____ 28. I am confident engaging with LGBTQ+ clients' around identifying and developing close bonds with supportive persons in their lives.
_____ 29. I am comfortable supporting LGBTQ+ persons in developing their own definitions of who constitutes family.
_____ 30. I am comfortable encouraging parents of LGBTQ+ persons to work through their feelings and accept and support their LGBTQ+ children as they are.
_____ 31. I can normalize an LGBTQ+ client's feelings during different points of the coming out process.
_____ 32. I can help LGBTQ+ clients navigate their lack of safety related to the coming out process.
_____ 33. I am confident that I can help transgender persons navigate healthcare policies and rulings.

Self-Efficacy Scoring Information

Add all 33 items above (ranges from 0 – 99) = ________

Interpretation: The higher your score, the more confident you are working with LGBTQ+ clients.

Copyright © 2023 Bradley O. Boekeloo, Jessica N. Fish, Elizabeth M. Aparicio, Richard Shin, Mia Smith-Bynum, Michael Vigorito, & Sean Lare. University of Maryland Prevention Research Center.

PRACTICE

Please indicate how often you do the following on a scale from:

0 = Never / Not applicable
1 = Almost never
2 = Sometimes
3 = Frequently
4 = All the time

_____ 1. I engage in a process of self-reflection to assess my own attitudes and emotions towards LGBTQ+ clients.
_____ 2. I stay current with the language used by LGBTQ+ people.
_____ 3. I stay connected with LGBTQ+ resources for professional development related to LGBTQ+ competency.
_____ 4. I help clients explore the meaning of their physical sexual drives, romantic attractions, and actual sexual behaviors when they are questioning their sexual orientation.
_____ 5. I help clients with gender dysphoria embrace their gender identity.
_____ 6. I support clients who want gender confirmation surgery in obtaining the affirmative healthcare that they need.
_____ 7. *I infer clients' sexual orientation based on their gender identity.
_____ 8. *I infer clients' gender identity based on their sexual orientation.
_____ 9. I use the name that my client uses regardless of their legal name.
_____ 10. I share my own pronouns when I introduce myself to clients.
_____ 11. I use the pronouns that my client uses.
_____ 12. I ask consent before using the sexual orientation and gender identity language that my client uses.
_____ 13. I ask consent before using any potentially sensitive language in reference to body parts and/or behaviors that my client uses.
_____ 14. When I meet a new client, I assess the pronouns and name they use.
_____ 15. I help LGBTQ+ clients identify and build on their strengths and resources.
_____ 16. I help LGBTQ+ clients identify their own internalized homophobia, biphobia, and/or transphobia.
_____ 17. I help clients identify external sources of internalized homophobia, biphobia, and/or transphobia.
_____ 18. I continually examine my own practice for ways that I might not be supportive to LGBTQ+ clients.
_____ 19. I try to speak up in my organization when I see things that might demean LGBTQ+ clients.
_____ 20. I assist my LGBTQ+ clients in obtaining behavioral, social, and medical services when appropriate.
_____ 21. I have made a list of behavioral, social, and medical services that are supportive of LGBTQ+ persons.
_____ 22. I do not assume that my LGBTQ+ clients are seeking mental health care because of concerns about their sexuality or gender.
_____ 23. When desired by LGBTQ+ clients, I explore the impact of racism on their mental health.
_____ 24. I engage with LGBTQ+ clients' around developing close bonds with supportive persons in their lives.
_____ 25. I encourage parents to work through their feelings and support their LGBTQ+ children as they are.
_____ 26. I normalize LGBTQ+ clients' feelings during different points of the coming out process.
_____ 27. I help LGBTQ+ clients navigate their lack of safety related to the coming out process.
_____ 28. I make a point of staying abreast of laws protecting LGBTQ+ rights in health care, employment, adoption, etc.

Practice Scoring Information

*Reverse code items 7 & 8 (where never = 4 and all the time/not applicable = 0)

Add all 28 items above (ranges from 0 – 112) = ________

Interpretation: The higher your score, the more often you practice LGBTQ+ affirming care.

TOTAL LGBTQ+ COMPETENCY SCORING INFORMATION

Add all 4 domains from above (grand total ranges from 0 – 311) = ________

Interpretation: The higher your score, the more competent you are in providing LGBTQ+ affirmative care.

Copyright © 2023 Bradley O. Boekeloo, Jessica N. Fish, Elizabeth M. Aparicio, Richard Shin, Mia Smith-Bynum, Michael Vigorito, & Sean Lare. University of Maryland Prevention Research Center.
